# Investigation of the impact of thirdhand e-cigarette exposure on platelet function: A pre-clinical study

**DOI:** 10.18332/tid/185286

**Published:** 2024-03-30

**Authors:** Shelby S. Umphres, Ahmed B. Alarabi, Hamdy E. A. Ali, Fadi T. Khasawneh, Fatima Z. Alshbool

**Affiliations:** 1 Department of Pharmaceutical Sciences, Irma Lerma Rangel School of Pharmacy, Texas A&M University, Kingsville, United States; 2 Department of Pharmacy Practice, Irma Lerma Rangel School of Pharmacy, Texas A&M University, Kingsville, United States

**Keywords:** e-cigarette, platelet, thrombosis, thirdhand tobacco exposure, cardiovascular disease

## Abstract

**INTRODUCTION:**

The use of e-cigarettes (ECs) has reached unprecedented levels, due to a variety of reasons, including the misconception regarding their safety. Thus, there have been efforts to characterize the effects of EC exposure, including in the context of thirdhand EC (THEC) on a host of disorders, such as cardiovascular disease (CVD).

**METHODS:**

To address this issue, we sought to characterize the effects of THEC on platelet function and thrombus formation, using a novel mouse exposure protocol that resembles real life scenarios. To assess these effects, a host of related *in vivo* (i.e. tail bleeding time, and ferric chloride injury induced thrombosis model) assays and *in vitro* platelet specific (e.g. aggregation, and dense granule secretion) investigative assays were conducted.

**RESULTS:**

Our *in vivo* characterization demonstrated that THEC exposed mice exhibited a prothrombotic phenotype reflected by their shortened tail bleeding (THEC: 37 ± 15 seconds, versus clean air: 183 ± 56 s) and occlusion times (THEC: 188 ± 39 s, versus clean air: 519 ± 70 s), relative to those exposed to clean air. Importantly, we found no difference in the platelet counts between the THEC and clean air mice. As for the underlying mechanism, separate experiments revealed significantly enhanced platelet aggregation, dense and alpha granule secretion, as well as integrin/GPIIb-IIIa activation and phosphatidylserine exposure in response to thrombin and ADP agonist stimulation.

**CONCLUSIONS:**

Taken together, these results provide evidence that THEC does have the capacity to increase the risk of thrombotic disease, which should increase awareness regarding its underappreciated negative health effects.

## INTRODUCTION

Cardiovascular disease (CVD) remains the leading cause of death, accounting for approximately 32% of total deaths worldwide^1,2^. Many of these disease states can be attributed to preventable risk factors, with the most common being cigarette smoking. Notably, thrombosis is the main mechanism by which smoking-related CVD can occur^3^, and sufficient evidence has shown that modulation/enhancement of platelet function is a major contributor to this phenotype. Although health risks associated with direct exposure to traditional smoking have been firmly established, it is noteworthy that there is emerging evidence suggesting that passive exposure does also pose a significant threat to non-smokers^4,5^. In this connection, secondhand smoke (SHS) exposure was found to increase the risk of CVD. Interestingly, there has been another form of exposure that has been recognized in the last decade or so, namely that to smoke residues that build up on surfaces after the smoke has been extinguished. This form of exposure – which is identified as thirdhand smoke (THS) – has also been found to be associated with a negative health impact. For example, THS – was shown to impair wound healing^6^ and enhance the risk of thrombosis^7^.

In recent years, there has been a rapid decline in the use of traditional cigarettes, seemingly due to the widespread knowledge and evidence-based recognition of their negative/public health effects. Electronic cigarettes (ECs) were introduced into the United States market in 2007, and their use has been rising at a rapid rate since then^1^. One of the underlying reasons for the popularity of these devices is related to their advertisement as a ‘safer alternative’ to traditional smoking, which, therefore, makes them appealing to a variety of users, including non-smokers, pregnant women, and youth^1,8,9^. With an assortment of modern and compact designs, and thousands of flavors to choose from^10^, these appealing aspects of ECs have been attracting users. However, these individuals are seemingly unaware of the harmful toxicants emitted by these devices, which can pose significant health threats. To this end, we have previously shown that direct EC exposure increases the risk of thrombotic CVD, via modulating platelet function^11,12^. Additionally, and interestingly, there is evidence that ECs are a source of thirdhand exposure (THEC) from the vapor^13,14^. Thus, these devices leave THEC residues that get embedded and accumulate on surfaces such as, carpets, couches, curtains, clothes, etc., whereby susceptible individuals unknowingly get exposed to^13,14^. Even though we have established a link between THS, including that which is in utero and occlusive CVD^7,15^, nothing is known in the context of THEC. Taken together, it is our aim to characterize the impact THEC exposure has on platelet function and thrombogenesis, using animal models.

## METHODS

### THEC exposure protocol

**Figure 1:**
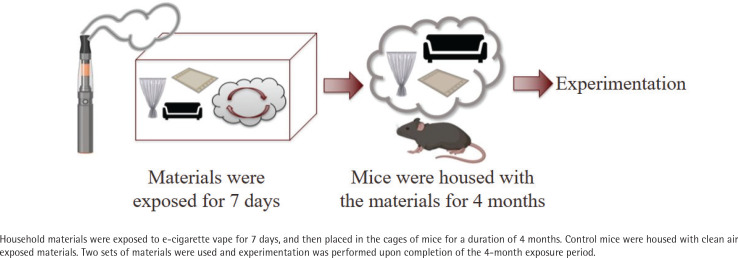
Depiction of the THEC exposure protocol

In order to mimic real-life exposure scenarios, the following THEC exposure protocol was developed and employed in part, based on the previously described THS protocol^7^. Household materials were exposed to EC vapor utilizing an e-vapeTM vapor inhalation system (La Jolla Alcohol Research, Inc). These materials, measured in inches, consisted of one piece (12 in × 3.5 in) fiber upholstery, three pieces (7.5 in × 5.5 in) cotton fabric, and two pieces (2 in × 2 in) fiber carpets and were exposed to 400 puffs per day with 3 s puff duration, with the airflow of the system maintained at 1 L/min. The device used to conduct these studies was a TFV8 Big Baby tank and ‘Absolute 0’ e-liquid containing 18 mg nicotine, 30/70 propylene glycol/vegetable glycerin (PG/VG), and menthol flavor. Male and female mice were utilized, which were allowed to live in cages that were furnished with the exposed materials for a duration of 4 months. Two sets were employed to allow for exchanging the material on a weekly basis with freshly exposed ones. The control mice were housed in cages furnished with material that was exposed to clean air (CA); referred to as CA-exposed mice. A simple depiction of the THEC exposure protocol is shown in Figure 1.

### Materials and reagents

ADP, thrombin, luciferase, and disposable materials such as stir bars and cuvettes were purchased from Chrono-Log Corporation (Havertown, PA, USA). APC anti-mouse CD41 and FITC conjugated P-selectin were purchased from BioLegend® (San Diego, CA, USA), JON/A Integrin αIIbβ3 from Emfret Analytics (Würzburg, Germany), FITC conjugated Annexin V from BD Biosciences (Franklin Lakes, NJ, USA). Apyrase and ferric chloride (FeCl_3_) were purchased from Sigma-Aldrich (St. Louis, MO, USA). PGI2 was purchased from Caymen Chemicals (Ann Arbor, MI, USA). E-liquid and the tanks were purchased from Vapor Chef (Bristol, PA, USA), and all materials used for exposure were purchased from Amazon. Filter papers were purchased from Fisher Scientific (Waltham, MA, USA) and all other reagents were of analytical grade.

### Animals

Male and female C57BL/6J mice were purchased from Jackson Laboratories (Bar Harbor, ME, USA) and housed in a group of 4 to 5 per cage. The mice were housed in standard conditions including 12/12 light/dark cycle, 24°C, with access to food and water ad libitum along with the material that was either exposed to THEC or CA. Mice were exposed to materials starting at 6 weeks of age, and experiments were performed immediately following the 4-month exposure period. All animal experimental protocols were approved by the Institutional Animal Care and Use Committee of Texas A&M University.

### Blood collection

Mice were anesthetized with 2–5% isoflurane using a SomnoFlow system, before blood was collected from the heart and combined with 50 µL of 3.8% the anticoagulant sodium citrate solution. Blood samples were spun at 180g for 12 min (Thermo Scientific Sorvall Legend XTR Centrifuge) and the platelet-rich plasma (PRP) was collected.

### Platelet and blood count

The platelet count was conducted using a HEMAVET® 950FS Multi-species Hematology System. The blood count for each individual mouse was also determined using this system.

### *In vitro* platelet aggregation

Blood was pooled from 5–8 THEC and CA-exposed mice, and the PRP was isolated before being activated with 0.05 U/mL of thrombin or 1 µM of the ADP agonist, and the aggregation response was measured using a model 700 Chrono-Log aggregometer. This experiment was repeated at least 3 times.

### Dense granule secretion

The PRP was incubated with 12.5 µL of the luciferase substrate, before being activated with 0.05 U/mL of thrombin or 1 µM of ADP, and the secretion response was measured using a model 700 Chrono-Log aggregometer.

### Washed platelet preparation

PRP was incubated with PGI2 (10 ng/mL) and apyrase (0.37 U/mL), before being spun at 400g for 10 min using a Thermo Scientific Sorvall Legend XTR Centrifuge. The supernatant was removed, and the pellet was resuspended in a mixture of HEPES/Tyrodes buffer (pH 6.5), PGI2 (10 ng/mL), apyrase (0.37 U/mL), and EGTA (0.5 M) before being spun once more at 400g for 10 min. The supernatant was removed, and the pellet was resuspended in HEPES/Tyrodes buffer (pH 7.4), and the platelets were left to rest for 30 min. Next, washed platelets were counted using the aforementioned hematology system, and counts were adjusted accordingly to ensure an equal number of platelets were used for both CA and THEC samples.

### Flow cytometric analysis

Washed platelets were incubated with APC anti-mouse CD41, and each of the following anti-mouse antibodies individually: JON/A Integrin αIIbβ3, FITC conjugated Annexin V, and FITC conjugated P-selectin for 15 min. Platelets were then activated with either 0.05 U/mL of thrombin or 1 µM of ADP for 5 min, and then diluted 2.5-fold with phosphate buffer saline (PBS). BD Accuri C6 and Cflow plus software (BD Biosciences) were used to measure the mean fluorescence intensity (MFI) and to analyze data, respectively.

### Tail bleeding time assay

To evaluate the hemostatic function of platelets, the tail bleeding assay was performed on both THEC and CA-exposed C57BL/6 mice, as previously described^7^. Measuring from the tip of the tail, a 5 mm segment was transected with a blade while the mice were under isoflurane anesthesia, and their temperature was monitored and maintained at 37^o^C with a homeothermic blanket. The tail was immediately submerged in 0.9% saline solution that was maintained at 37^o^C, and the time until the tail bleeding stopped was measured^16^. For the purpose of statistical analysis, the cutoff time was considered to be 10 min in order to prevent excessive bleeding.

### Ferric chloride-induced carotid artery thrombosis model

Mice from the two exposure groups were injected with avertin to anesthetize them prior to the left carotid artery being isolated and cleaned. Next, a 0.5 mm micro-flow probe (Transonic Systems Inc.) was used to measure the baseline blood flow for 1 min and ensure stability. A 1 mm diameter disc filter paper with 7.5% ferric chloride was then placed on the artery for 3 min to induce injury. The time to occlusion was measured immediately after the filter paper was removed. For statistical analysis, a 15-min occlusion time was considered to be the cutoff time^11^.

### Statistical analysis

Experiments such as tail bleeding time and the ferric chloride thrombosis model were performed on individual living mice and all others were performed using pooled blood from at least 5 mice and repeated at least three times. GraphPad PRISM (San Diego, CA, USA) statistical software (version 7.0) was used for data analysis and results are presented as mean ± SD. A normality test was done for all data, and based on these results, parametric tests were used, when the data were normally distributed, and non-parametric tests were used when the data were not normally distributed. Thus, the occlusion time, bleeding time, aggregation, and blood counts data were analyzed with t-test, and the flow cytometry data was analyzed with one-way ANOVA. All tests performed were two-tailed, and statistical significance was accepted at p<0.05.

## RESULTS

### Exposure to THEC enhances platelet aggregation and dense granule secretion in response to agonist stimulation

**Figure 2:**
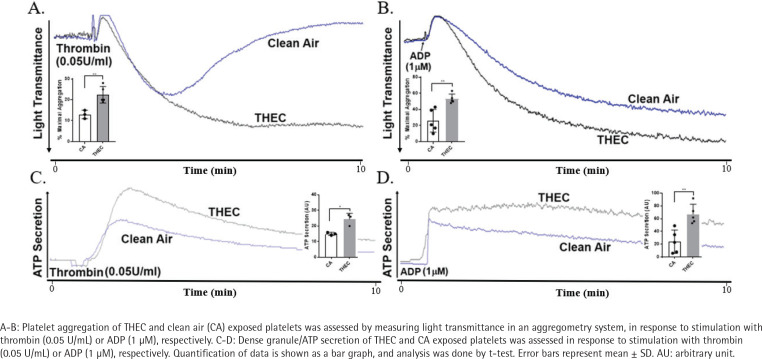
THEC exposed platelets show enhanced aggregation and dense granule secretion in response to agonist stimulation, in comparison to CA controls

To assess the effect of THEC on (*in vitro*) platelet function – following a 4-month exposure period – aggregation response was evaluated. These data showed that as a result of THEC exposure, platelets exhibited a more hyperactive aggregation state in comparison to those exposed to CA, when stimulated with either thrombin (Figure 2A) or ADP (Figure 2B). Additionally, dense granule secretion was also found to be increased in the THEC-exposed platelet when stimulated with thrombin (Figure 2C) or ADP (Figure 2D).

### Exposure to THEC enhances α-Granule secretion, integrin αIIbβ3 activation and phosphatidylserine exposure in response to agonist stimulation

**Figure 3:**
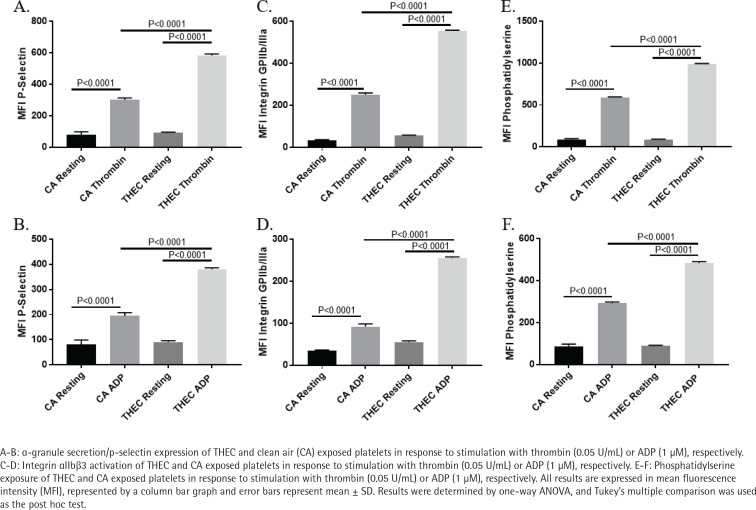
THEC exposed platelets show enhanced α-granule secretion, integrin αIIbβ3 activation and phosphatidylserine exposure responses in response to agonist stimulation

Though our data revealed an enhanced aggregation and dense granule secretion phenotype in the THEC-exposed platelets, we sought to further analyze its impact on platelet function by determining the effects on separate activation markers, starting with α-granule secretion. Thus, when platelets were stimulated with either thrombin (0.05 U/mL) or ADP (1 µM), their surface p-selectin levels (measured using flow cytometry) were found to be significantly more in those that were exposed to THEC (Figures 3A and 3B). Moreover, and similarly, platelet integrin αIIbβa3 activation and phosphatidylserine exposure also showed a significant enhancement in response to thrombin (0.05 U/mL) or ADP (1 µM) under THEC exposure conditions (Figures 3C–3F). These data are consistent with the aggregation and dense granule secretion findings and indicate that after a 4-month exposure, THEC-exposed platelets are in a more hyperactive state.

### Exposure to THEC enhances hemostasis

**Figure 4:**
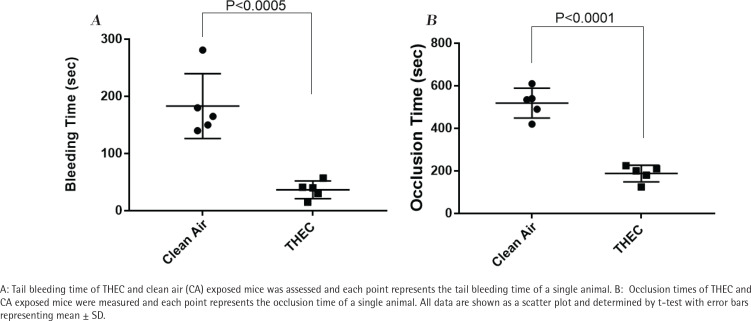
THEC exposure significantly shortens tail bleeding time and occlusion time in tail bleeding time assay and ferric chloride carotid artery injury-induced thrombosis model

Based on the *in vitro* results observed thus far – namely enhanced platelet function – we next sought to determine if the ‘same’ phenotype would be observed *in vivo*, which was addressed by conducting the tail bleeding time assay. As shown in Figure 4, significantly shortened bleeding times were observed in THEC-exposed mice in comparison to the CA, namely 37 ± 15 s versus 183 ± 56 s, respectively. These data, again, are consistent with the hyperactive state of the THEC-exposed mice platelets and suggest increased susceptibility for developing occlusive CVD.

### Exposure to THEC enhances thrombus formation

Altered physiological hemostasis can suggest that the exposed mice have the potential to be more susceptible to thrombus formation. Consequently, we sought to further characterize the impact of our exposure conditions (THEC vs CA) *in vivo* using the FeCl_3_ carotid artery injury-induced thrombosis model to measure the occlusion times. As one might expect in light of the effects observed, there was a significant decrease in the occlusion time of the THEC exposed mice in comparison to the CA, namely 188 ± 39 s versus 519 ± 70 s, respectively (Figure 4), which indicates a prothrombotic phenotype and an increased risk for occlusive CVD/thrombogenesis.

### THEC exposure does not impact blood cell count

In order to eliminate the possibility that the observed phenotype was impacted – at least in part – by changes in the number of platelets or blood cells, the counts for each individual mouse were taken. No significant differences were observed in any cell type examined (Table 1).

**Table 1:** Blood cell counts of individual THEC and CA exposed mice

Cell type	CA Mean ± SD	THEC Mean ± SD	p
Platelets (k/μL)	539.5 ± 19.43	528.7 ± 34.37	0.789
Mean platelet volume (fL)	4.133 ± 0.04	4.017 ± 0.10	0.313
Red blood cells (M/μL)	8.493 ± 0.45	7.683 ± 0.70	0.353
Lymphocytes (k/μL)	3.438 ± 0.26	2.742 ± 0.23	0.0723
HCT (%)	41.42 ± 2.18	36.67 ± 3.34	0.261
Monocytes (k/μL)	0.25 ± 0.02	0.21 ± 0.03	0.243
White blood cells (k/μL)	4.053 ± 0.32	3.233 ± 0.29	0.088

Blood from individual THEC and CA exposed mice was collected, before the blood cells were counted as described in the methods sections. These data indicate that there were no significant differences in blood cell counts between THEC and CA exposed mice. Blood cell count analyses were conducted using t-test.

## DISCUSSION

In this study, and for the first time, we have evaluated and reported the consequences of 4 months of THEC exposure in the context of platelet function and occlusive CVD. Collectively, our findings indicate that THEC exposure may lead to platelet hyperactivity, namely enhanced aggregation, secretion, integrin activation and phosphatidylserine exposure, and that is accompanied by an enhanced risk of cardiovascular occlusive diseases. The hypothesis driving the present work is that THEC modulates platelet activation and thrombogenesis. In order to address this hypothesis, we utilized a well-established exposure protocol to help close knowledge gaps regarding THEC, employing a real-life exposure scenario. The latter involved using household items, namely carpets, curtains, couches, etc.^12^, as well as 18 mg nicotine, 30/70 PG/VG, and menthol flavor EC vape, shown to be one of the most popular flavor/nicotine combinations^12^. In our *in vitro* studies, a significant increase in the platelet aggregation response was observed in THEC-exposed platelets, when compared to CA, in response to agonist stimulation, namely ADP and thrombin. Moreover, dense and alpha (α) granule secretion also showed an increase in the THEC-exposed platelets. These data indicate that the THEC-exposed platelets are in a hyperactive state in response to agonist stimulation, suggesting that individuals exposed to this form of smoke would be at an increased risk for CVD. While these effects appear to be consistent with what we have seen with THS^7,15^, future studies will focus on directly comparing the effects of THEC and THS. In this connection, a recent study^17^, compared the toxicant profile from both, indicating that these forms of exposure do share some common toxicants, but they are not identical. Though the e-liquid is noted to be less complex, this study highlighted the variety of chemicals that can be formed based on the multitude of settings that these devices can accommodate, and even more of a concern when they become heated or undergo chemical reactions in the form of THEC. This further underscores the notion that the negative health impact of THEC warrants further investigation.

To determine if the negative health effects of THEC could also be seen under *in vivo* settings, the tail bleeding time and the carotid artery injury-induced thrombosis model were performed. These data, again, revealed a significantly enhanced (shortened) bleeding and thrombosis occlusion times in the exposed mice, relative to the CA-exposed controls. These findings are consistent with *in vitro* (e.g. aggregation) data and provide additional evidence that THEC not only modulates hemostasis but also has the potential to increase the risk of occlusive CVD. These data uncover a previously unidentified/underestimated risk for the development of thrombotic CVD in mice, which opens an unexplored research area that needs to be pursued further, especially in humans. To further investigate the platelet phenotype under THEC exposure conditions, we assessed integrin αIIbβa3 activation and phosphatidylserine exposure. Indeed, when stimulated with agonists, we again saw a significant increase in the aforementioned responses in the THEC-exposed platelets, which is consistent with the phenotype observed thus far. The enhanced phenotype observed in THEC-exposed platelets with regard to phosphatidylserine expression is of significant importance, given its role in the activation of the coagulation cascade^18^. Of note, it is important to emphasize that THEC exposure did not modulate platelet count, at least under the present experimental conditions, and hence all observed phenotypes can be attributed to the exposure conditions. Alarmingly and last but not least, these results document that even thirdhand exposures that are of e-cigarette origin (THEC), which are devices claimed to be ‘safer’ than traditional cigarettes, can exert detrimental CV health effects. This is important given that the use of ECs has been on the rise more prominently in the past few years^19^. In future studies, we will evaluate the detailed mechanism by which the THEC modulation of platelet function contributes to increased risk for CVD, in the context of sex.

Due to the known morbidity and mortality related to CVDs, it is of the utmost importance to identify unknown risk factors or environmental contributors that predispose to such diseases. As previously noted, cigarette smoking is the most established preventable risk factor that leads to CVD. In addition, several reports have shown that e-cigarettes are associated with negative effects that could lead to a higher risk of heart attacks and coronary heart disease^3,19^. Parallel to these findings, there is evidence that the dual use of ECs with traditional cigarettes increases the risk for stroke^19^, and endothelial cell dysfunction and oxidative stress, with the latter also associated with prothrombotic cardiovascular events^11,20,21^. While growing evidence has demonstrated the association between direct exposure to ECs and CVD^11,12^, there is a gap in knowledge concerning the health impact of indirect exposure to ECs, including thirdhand exposure (THEC), especially in the context of occlusive CVD, an area in which virtually nothing is known. However, a few or none involved platelets, recent studies did highlight THEC effects in other systems. To this end, a recent study by Thorpe et al.^22^ utilized a somewhat similar exposure protocol and documented that THEC without nicotine caused significant damage to the pulmonary system. Other studies have suggested that THEC exposure alters organ development and immune responsiveness^23,24^. To this end, it is important to note the interconnectedness between the pulmonary and cardiovascular systems, as damage to one could indirectly impact the other, leading to several health complications. It is also important to note that nicotine does not appear to be the only ‘toxicant’ underlying the negative health effects of THEC. The complexity of these devices adds challenges when studying their effects, due to a host of factors, including the variety of device settings the users can select, amongst others^25^. Thus, the levels of heat and voltage that can be applied allow the e-liquid to heat at different temperatures, thereby potentiating the production of hundreds of different chemicals^25^, all of which have the potential to increase the deleterious health effects of ECs and/or THEC.

We have previously utilized a validated thirdhand smoke (THS) mouse model and shown that THS exposure modulates platelet function both *in vitro* and *in vivo* ^7,26^; however, whether THEC – whose chemicals vary in comparison to THS^17^ – produces similar/any effects is yet to be studied. Though a direct comparison has not been made between the two forms of thirdhand exposure, it is important to highlight some of our findings. In the THS study, it was found that after three months of exposure to 40 cigarettes, a significant phenotype was observed, in which there was a significant increase in platelet hyperactivity^7^. Our separate ongoing studies appear to indicate that even when the exposures are for only one month, THS can still increase the risk of thrombosis. To this end, the THEC studies that were done in a manner identical to the present study, but for a duration of only two months, did not – interestingly– reveal any significant differences with regard to the effects on platelets in comparison with CA.

### Limitations

There are limitations to this study. Though our study has provided critical insight regarding the impact of THEC on occlusive CVD, our study was not designed to characterize the detailed mechanism by which it exerts its effects. We also acknowledge that we utilized a high number of puffs in order to simulate a heavy user or multiple users in the same room. Furthermore, our study was limited to mice, and despite the fact that they are commonly used in tobacco exposure studies, one cannot exclude differences between mice and humans.

## CONCLUSIONS

Our results show that THEC exposure has significant effects on platelet function both *in vitro* and *in vivo*, indicating the potential for an increased risk for thrombosis-based CVD. In future studies, we aim to investigate whether this phenotype manifests in a sex-dependent manner and also further explore the time-dependent effects of THEC exposure.

## Data Availability

The data supporting this research are available from the authors on reasonable request.
